# Effectiveness of Sucrose Versus Breast Milk as Non-Pharmacological Measures in the Management of Neonatal Pain: A Systematic Review

**DOI:** 10.3390/children13050676

**Published:** 2026-05-14

**Authors:** Marina Camacho-Pernil, Pastora Tirado-Hernández, María Rodríguez-García, Elena Andrade-Gómez, Javier Fagundo-Rivera, Pablo Fernández-León

**Affiliations:** 1 Department of Surgery and Internal Medicine, Hospital Universitario Vall d’Hebron, Catalonia Health Service, 08035 Barcelona, Spain; 2 Department of Surgery and Internal Medicine, Hospital of Calahorra, Rioja Health Service, 26500 La Rioja, Spain; 3 Centro Universitario de Enfermería Cruz Roja, University of Seville, 41009 Seville, Spain; 4 Pre-Departmental Unit of Biomedical Sciences and Health Specialties, University of La Rioja, 26004 Logroño, Spain; 5 Department of Sociology, Social Work and Public Health, Faculty of Labour Sciences, University of Huelva, 21007 Huelva, Spain; 6 Department of Nursing, Faculty of Nursing, Physiotherapy and Podiatry, University of Seville, 41009 Seville, Spain

**Keywords:** neonatal pain, neonate, non-pharmacological interventions, sucrose, breast milk, breastfeeding, pain management, neonatal intensive care unit, randomized controlled trials, procedural pain

## Abstract

**Highlights:**

**What are the main findings?**

- Both sucrose and breast milk significantly reduce neonatal pain during common clinical procedures.
- Multimodal approaches combining these interventions with techniques such as kangaroo care or non-nutritive sucking enhance analgesic effects.

**What are the implications of the main findings?**

- These interventions provide safe, low-cost, and effective alternatives to pharmacological analgesia in neonatal care.
- Their integration into standardized clinical protocols can improve pain management and promote humanized, family-centered care.

**Abstract:**

**Background**: The neonatal period involves rapid physiological adaptation and high vulnerability to painful stimuli, especially in NICU-admitted infants. Neonates have the neurophysiological capacity for nociception, and repeated pain exposure may impair neurodevelopment. Non-pharmacological interventions, particularly oral sucrose and breast milk, are widely used as first-line analgesic strategies due to their safety and efficacy. However, heterogeneity in existing studies requires evidence synthesis. **Methods**: A systematic review following PRISMA guidelines was conducted to assess the effectiveness of sucrose and breast milk in neonatal pain reduction. PubMed, Scopus, CINAHL, and Web of Science were searched for randomized controlled trials published between 2019 and 2024. Studies involving neonates undergoing painful procedures and receiving sucrose, breast milk, or both were included. Data extraction and risk of bias assessment were performed independently. Due to heterogeneity in interventions and outcomes, a narrative synthesis was conducted. **Results**: Thirteen randomized controlled trials were included. Both sucrose and breast milk consistently reduced neonatal pain scores and physiological indicators such as heart rate and oxygen saturation. Sucrose showed rapid, short-term analgesia mediated by endogenous opioid pathways, while breast milk provided additional sensory, nutritional, and emotional benefits that support mother–infant bonding. Multimodal approaches, including kangaroo care, non-nutritive sucking, and swaddling, enhanced analgesic effects. Heterogeneity in protocols and assessment tools limited comparability across studies. **Conclusions**: Sucrose and breast milk are safe and effective non-pharmacological interventions for neonatal pain management. Their incorporation into standardized multimodal protocols is recommended to optimize analgesia and promote humanized neonatal care. Further research is needed to standardize dosing and evaluate long-term outcomes.

## 1. Introduction

The neonatal period, defined by the World Health Organization as the interval from birth to 28 days of life, represents a critical developmental stage characterized by profound physiological adaptations that enable the transition to extrauterine life [1]. During this period, newborn infants are highly vulnerable. Those born preterm or with low birth weight are particularly at risk and frequently require admission to neonatal intensive care units (NICUs). In these units, they are exposed to multiple diagnostic and therapeutic procedures [2].

Within this context, neonatal pain has emerged as a clinically significant concern. Pain is currently recognized as the fifth vital sign and is defined as an unpleasant sensory and emotional experience associated with actual or potential tissue damage [3]. Contrary to earlier assumptions that underestimated neonatal pain perception, robust evidence demonstrates that neonates possess the requisite anatomical, neurophysiological, and hormonal substrates for nociception even before birth [4,5]. Moreover, repeated exposure to painful stimuli has been associated with immediate physiological, biochemical, and behavioral alterations, as well as potential long-term adverse effects on neurodevelopment and stress regulation [6].

Pain assessment in neonates relies on validated instruments based on physiological and behavioral indicators, such as Crying, Requires O_2_, Increased vitals, Expression, Sleeplessness (CRIES), Premature Infant Pain Profile (PIPP and PIPP-R), and Neonatal Infant Pain Scale (NIPS), which enable systematic and objective evaluation [7]. However, beyond accurate assessment, the implementation of effective pain management strategies remains essential.

There is increasing interest in non-pharmacological interventions as first-line approaches due to their favorable safety profile, low cost, and absence of significant adverse effects. These strategies encompass environmental, behavioral, and nutritional interventions, including non-nutritive sucking, kangaroo care, sensory stimulation, and oral sweet solutions [8]. Among these, sucrose or glucose administration and breastfeeding are particularly noteworthy due to their well-documented analgesic efficacy [9].

Sucrose, typically administered as a 24% oral solution, is one of the most extensively studied interventions for neonatal pain management. Its analgesic effect is thought to be mediated by the release of endogenous opioids following activation of sweet taste receptors, thereby attenuating both physiological and behavioral responses to pain [10]. Furthermore, its efficacy may be enhanced when combined with other interventions, such as non-nutritive sucking, resulting in a synergistic effect [11]. In addition to opioid-mediated pathways, emerging evidence suggests that sucrose may also exert calming or sedative effects, potentially contributing to the reduction in observable pain-related behaviors independently of a direct analgesic action [12]. Importantly, some of these mechanistic insights derive from translational studies conducted in rodent models, which should be considered when extrapolating findings to human neonates [12].

Breast milk, in contrast, represents a natural analgesic intervention with a multifactorial mechanism of action. Its effects are not only attributable to its biochemical composition, mainly lactose, tryptophan, and opioid peptides, but also to associated factors such as skin-to-skin contact, maternal–infant bonding, and multisensory stimulation during breastfeeding [13]. Several studies have demonstrated its capacity to attenuate pain responses, even in the absence of direct sucking [14].

Despite the growing body of evidence, published studies exhibit considerable heterogeneity in both outcomes and pain assessment methodologies. Recent systematic reviews have confirmed the effectiveness of these interventions in reducing neonatal pain scores; however, they also highlight the need for standardized assessment tools and further investigation into long-term outcomes and specific populations, such as preterm infants [15,16]. Additionally, some authors caution that reductions in pain scale scores may not fully capture the primary nociceptive experience, emphasizing the need for further research in this area [15].

From a clinical and caregiving perspective, this issue is particularly relevant given that hospitalized neonates may experience numerous painful procedures during their first days of life, with potential implications for neurological development and overall well-being [17]. Furthermore, the implementation of standardized neonatal pain management protocols remains limited in certain settings, including Spain, where evidence suggests suboptimal use of analgesic measures during routine procedures [18].

In this context, strengthening the evidence base for effective, safe, and easily implementable non-pharmacological interventions is essential to improving the quality of neonatal care and promoting a more humanized approach [19].

Against this background, the present study aims to evaluate the effectiveness of sucrose and breast milk as non-pharmacological strategies for neonatal pain management. Specifically, it seeks to assess their clinical utility, characterize their individual effects on pain responses, and compare their efficacy both as standalone interventions and in combination with other non-pharmacological approaches in the neonatal population.

## 2. Materials and Methods

A systematic review of the literature was conducted to identify and synthesize studies evaluating the effectiveness of sucrose and breast milk as non-pharmacological interventions for neonatal pain management.

### 2.1. Search Methodology

A PICO framework was used to guide the review process, defining the population as neonates (P), the intervention as sucrose administration (I), the comparator as breast milk (C), and the outcome as pain reduction (O). Based on this structure, the following research question was formulated: Are sucrose and breastfeeding effective in reducing pain in neonates undergoing painful procedures?

This approach enabled a focused and systematic identification of relevant studies assessing commonly used non-pharmacological strategies for neonatal pain management. The literature search was conducted in PubMed, Scopus, CINAHL, and Web of Science (WOS) between 31 March and 9 April 2026. Detailed search strategies, applied filters, and results are presented in Table 1.

### 2.2. Selection Criteria

Studies were included if they met the following criteria: randomized controlled trials (RCTs) involving neonates, published between 2019 and 2026, and evaluating the effect of sucrose, breast milk, or their combination on neonatal pain reduction. Exclusion criteria comprised systematic reviews, meta-analyses, cross-sectional, observational, and cohort studies, pilot studies, studies not specifically involving neonatal populations, and those not assessing the analgesic efficacy of sucrose or breast milk.

### 2.3. Sources of Information

The primary data sources were PubMed, Scopus, CINAHL, and WOS. The review protocol was registered in PROSPERO under the identifier CRD420261361733.

As this study is a systematic review that did not involve human participants or individual-level data, ethical approval from an institutional review board was not required.

### 2.4. Search Strategy

The search strategy combined the following terms: (sucrose OR breastfeeding OR breastfed OR “breast milk”) AND (pain OR “physical suffering” OR ache*) AND (infant* OR neonate* OR newborn*).

### 2.5. Selection Process

Study selection followed the Preferred Reporting Items for Systematic Reviews and Meta-Analyses (PRISMA) guidelines [20]. All retrieved records were imported into Zotero reference manager version 9.0.3 (Corporation for Digital Scholarship, Falls Church, VA, USA) for reference management and duplicate removal.

Two reviewers independently screened titles and abstracts according to the eligibility criteria. Full-text articles were subsequently assessed for inclusion. Discrepancies were resolved through discussion and, when necessary, consultation with a third reviewer.

### 2.6. Data Extraction Process

Data extraction was performed independently by two reviewers using a standardized data collection form. Extracted variables included study characteristics, participant details, interventions, comparators, outcomes, and principal findings. Disagreements were resolved by consensus or consultation with a third reviewer. When relevant data were missing or unclear, study authors were contacted via email for clarification.

### 2.7. Study Variables

The exposure variables were oral sucrose and breast milk administered as non-pharmacological analgesic interventions. The primary outcome was pain reduction, assessed through changes in physiological parameters and behavioral responses, using validated neonatal pain assessment scales to ensure measurement objectivity.

### 2.8. Assessment of Methodological Quality

The methodological quality of the included studies was assessed using the Joanna Briggs Institute (JBI) Critical Appraisal Checklist for Randomized Controlled Trials [21]. A 13-item checklist was applied, addressing key methodological domains, including randomization procedures, allocation concealment, blinding (participants, personnel, and outcome assessors), baseline comparability, completeness of follow-up, reliability of outcome measurement, adherence to interventions, appropriateness of statistical analyses, and overall study design.

Two reviewers independently assessed each study, assigning judgments of “Yes,” “No,” “Unclear,” or “Not applicable” for each item. Discrepancies were resolved through discussion or, if necessary, adjudication by a third reviewer (Table 2).

### 2.9. Assessment of the Risk of Bias

**Figure 1:**
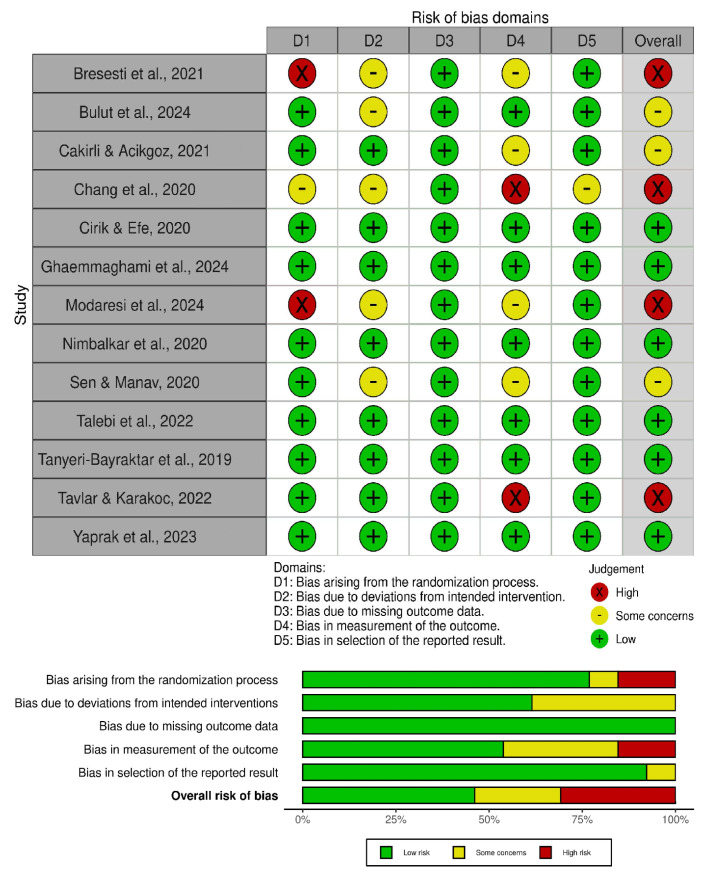
Summary of Risk of Bias assessment using ROB-2 Tool [6,10,22,23,24,25,26,27,28,29,30,31,32].

The methodological quality of the included studies was assessed using the Cochrane Risk of Bias 2.0 (RoB-2) tool for randomized controlled trials [33]. This tool evaluates five methodological domains: bias arising from the randomization process, deviations from intended interventions, missing outcome data, measurement of outcomes, and selection of the reported results. Each domain was classified as “low risk,” “some concerns,” or “high risk” based on the information provided in the studies. An overall risk-of-bias judgment was subsequently assigned for each study according to the RoB-2 guidance, considering the combined assessment across all domains. Studies were classified as low risk when all domains were judged as low risk, as having some concerns when at least one domain raised concerns without any high-risk judgments, and as high risk when one or more domains were rated as high risk or when multiple domains presented some concerns that could substantially lower confidence in the results (Table 3; Figure 1).

### 2.10. Data Synthesis

The characteristics of the included studies (population, interventions, comparators, and outcomes) were systematically tabulated to facilitate assessment of eligibility and comparability. Studies were grouped according to outcome measures, and data were standardized where necessary to ensure consistency in presentation. Results are reported in structured tables.

Following detailed evaluation of the 13 included randomized controlled trials, quantitative synthesis was deemed inappropriate due to substantial clinical and methodological heterogeneity. Notable variability was observed in intervention protocols (including differing formulations and dosages of sucrose and breast milk), types of painful procedures, outcome measures, timing of assessments, and population characteristics.

An exploratory subgroup meta-analysis was considered using standardized mean differences for the following comparisons: sucrose versus breastfeeding, sucrose versus control or no intervention, and breast milk (in its various forms) versus control. However, heterogeneity in study design, measurement instruments, and intervention protocols precluded valid statistical pooling.

Specifically, for the direct comparison between breastfeeding and sucrose, only two studies met the inclusion criteria, with discrepancies in pain assessment scales and inconsistent findings. Furthermore, one study did not comprehensively report means and standard deviations, limiting the calculation of standardized effect sizes and preventing quantitative synthesis. The limited data availability, combined with discordant effect directions, would likely have resulted in substantial heterogeneity and reduced the validity of any meta-analysis.

Accordingly, a meta-analysis was not performed. Instead, a narrative synthesis was performed, allowing for a more robust and context-sensitive interpretation of the available evidence, particularly in light of the identified heterogeneity across studies.

## 3. Results

### 3.1. Study Selection

The literature search conducted in PubMed, Scopus, CINAHL, and WOS between 31 March and 9 April 2026, using the predefined strategy and filters, identified a total of 3712 records.

Following duplicate removal using the Zotero reference manager version 9.0.3 (Corporation for Digital Scholarship, Falls Church, VA, USA) (*n* = 3197), 515 unique articles remained and were screened based on titles and abstracts. Of these, 390 studies were excluded for not meeting the inclusion criteria.

**Figure 2:**
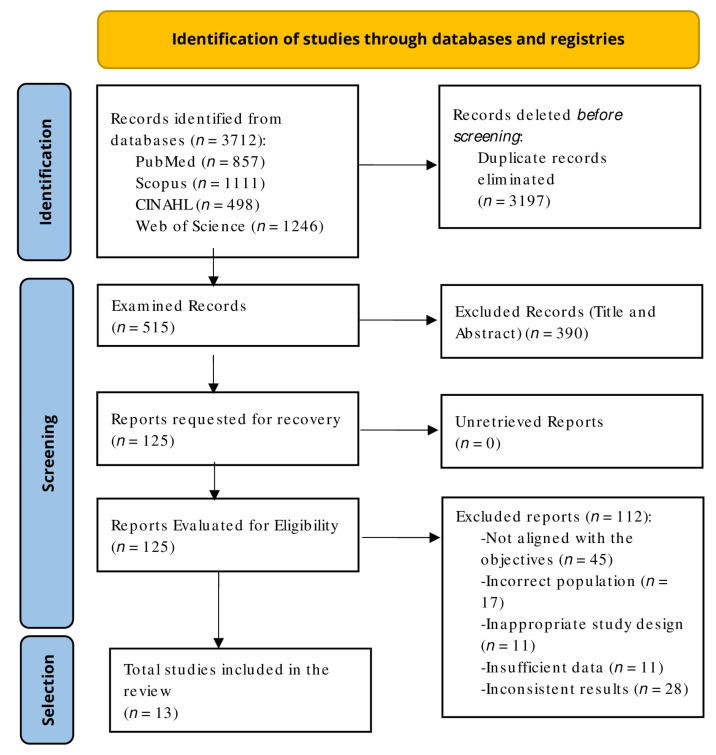
PRISMA flowchart.

A total of 125 full-text articles were subsequently assessed for eligibility, of which 13 met the inclusion criteria and were included in the review. The remaining 112 studies were excluded for specific reasons detailed in Figure 2.

### 3.2. Characteristics of the Included Studies

All 13 included studies were prospective randomized controlled trials published between 2019 and 2024 [6,10,22,23,24,25,26,27,28,29,30,31,32]. The temporal distribution was as follows: 2019 (*n =* 1; 7.7%), 2020 (*n =* 4; 30.8%), 2021 (*n =* 2; 15.4%), 2022 (*n =* 2; 15.4%), 2023 (*n =* 1; 7.7%), and 2024 (*n =* 3; 23.1%).

In terms of geographical distribution, most studies were conducted in Turkey (*n =* 7; 53.8%), followed by Iran (*n =* 3; 23.1%), the United States (*n =* 1; 7.7%), Italy (*n =* 1; 7.7%), and India (*n =* 1; 7.7%). Sample sizes ranged from 54 to 226 neonates, generally with homogeneous baseline characteristics, facilitating comparability across studies. All articles were published in English.

Pain outcomes were assessed using validated neonatal pain scales based on physiological and behavioral indicators observed during painful procedures. In all studies, nursing staff were responsible for pain assessment and administration of analgesic interventions. The most frequently used instruments included the Neonatal Infant Pain Scale (NIPS), the Assessment of Pain in Neonates Scale (ALS-Neo), the Premature Infant Pain Profile (PIPP), the Bernese Pain Scale for Neonates (BPSN), the Neonatal Infant Acute Pain Assessment Scale (NIAPAS), and the Neonatal Pain, Agitation and Sedation Scale (N-PASS). These tools evaluate parameters such as facial expression, crying, posture, respiratory pattern, oxygen saturation, and heart rate; notably, the N-PASS also incorporates agitation and sedation, providing a more comprehensive assessment of neonatal well-being.

The methodological quality of the included RCTs was evaluated using the Joanna Briggs Institute (JBI) Critical Appraisal Checklist consisting of 13 items. Overall, most studies demonstrated adequate baseline comparability, outcome measurement, follow-up completeness, and statistical analysis. Common limitations were related to randomization procedures, allocation concealment, and blinding of participants, personnel, and outcome assessors. Studies such as Cirik and Efe [25], Nimbalkar et al. [28], Talebi et al. [10], and Yaprak et al. [32] demonstrated high methodological quality (12/13), whereas Bresesti et al. [6], Chang et al. [24], and Modaresi et al. [27] achieved lower scores (6–7/13), primarily due to insufficient reporting or absence of randomization and blinding procedures. Overall, although several trials were methodologically robust, potential risks of selection and performance bias should be considered when interpreting the findings (Table 2).

Out of the 13 included studies, six were judged to have a low risk of bias across all ROB-2 domains, including Cirik and Efe [25], Ghaemmaghami et al. [26], Nimbalkar et al. [28], Talebi et al. [10], Tanyeri-Bayraktar et al. [30], and Yaprak et al. [32]. These studies generally demonstrated robust randomization procedures, appropriate allocation concealment, minimal or no missing outcome data, blinded outcome assessment, and the use of validated neonatal pain scales such as PIPP, PIPP-R, NIPS, and BPSN. In addition, several studies reported prior trial registration and standardized outcome assessment protocols, further strengthening methodological transparency and reliability.

Four studies, Bulut et al. [22], Cakirli and Acikgoz [23], Sen and Manav [29], and Chang et al. [24], were judged as presenting some concerns. The main limitations in these studies were related to the impossibility of blinding personnel delivering the interventions or uncertainty regarding the blinding of outcome assessors in studies relying on subjective pain measurements. In Chang et al. [24], additional concerns arose from the non-random recruitment of the control group and retrospective trial registration.

The remaining three studies, Bresesti et al. [6], Modaresi et al. [27], and Tavlar and Karakoc [31], were assessed as having a high risk of bias. The primary reasons included inadequate or predictable randomization methods, lack of allocation concealment, and absence of blinded outcome assessment despite the use of subjective neonatal pain scales. These methodological limitations may have influenced the estimation of treatment effects.

Overall, the methodological quality of the included studies was considered acceptable. Most trials showed adequate management of missing data, balanced baseline characteristics, and consistent use of validated pain assessment instruments. Although blinding was frequently challenging due to the nature of non-pharmacological interventions, the majority of studies implemented strategies to minimize detection bias, supporting the overall reliability of the findings synthesized in this review (Table 3; Figure 1).

Additional study characteristics and outcomes are summarized in Table 4 and Table 5.

### 3.3. Utility of Sucrose and Breast Milk in Neonatal Pain Management

Five randomized controlled trials evaluated the comparative efficacy of sucrose and breast milk in neonatal pain management [6,26,27,28,29]. These studies primarily involved neonates, particularly preterm infants, undergoing common painful procedures in neonatal intensive care settings, such as venipuncture, and emphasized the role of non-pharmacological strategies, including kangaroo care and sucrose administration.

Three studies [26,28,29] combined kangaroo care with the administration of 24% oral sucrose. Skin-to-skin contact was shown to stabilize physiological parameters, including heart rate, oxygen saturation, and crying duration, while also improving thermoregulation, facilitating breastfeeding initiation, and strengthening mother–infant bonding. These findings support kangaroo care as a cornerstone intervention in neonatal care. However, sucrose demonstrated a more pronounced analgesic effect, likely mediated by activation of sweet taste receptors and subsequent endogenous opioid release. Its calming effect lasted approximately 10 min, with peak efficacy observed within the first two minutes following administration.

Bresesti et al. [6] compared breastfeeding, liquid sucrose, and 24% sucrose gel, reporting comparable efficacy across all three interventions in reducing pain responses. Breastfeeding provided additional physical and emotional benefits, whereas sucrose gel was identified as a practical alternative when breastfeeding was not feasible. The increased sucking effort associated with gel administration may enhance parasympathetic activation and oxytocin release, thereby contributing to improved analgesia.

Modaresi et al. [27] investigated the analgesic effects of breast milk-related sensory stimuli, specifically olfactory and gustatory inputs. At 30 s post-procedure, olfactory stimulation produced greater pain reduction, whereas at 60 s, gustatory stimulation demonstrated superior efficacy. These findings suggest that breast milk-associated sensory cues may provide analgesic effects comparable to sucrose, highlighting the importance of maternal sensory stimuli in neonatal pain modulation.

Two studies [30,32] focused exclusively on sucrose, examining optimal dosing and administration frequency. Tanyeri-Bayraktar et al. [30] identified 0.2 mL/kg of 24% sucrose as the minimum effective dose for neonates up to one month of age during painful procedures. Yaprak et al. [32] reported that omission of the conventional two-minute interval between doses did not significantly affect immediate analgesic efficacy, although maximal effect was observed at 2–3 min. Both studies point out the need for further research to refine dosing strategies and administration protocols.

Regarding breast milk, three trials [22,23,31] evaluated its effectiveness as a non-pharmacological analgesic intervention. Tavlar and Karakoc [31] compared breastfeeding, breast milk odor, and maternal heartbeat sounds, finding that breastfeeding and auditory maternal stimuli were the most effective in reducing pain and stress. Cakirli and Acikgoz [23] demonstrated that exposure to the neonate’s own mother’s breast milk odor was more effective than that of another mother, emphasizing the role of olfactory recognition in early sensory processing and pain modulation. Bulut et al. [22] extended these findings by incorporating neurophysiological measures, showing that breast milk reduced cortical pain responses and shortened crying duration, supporting its analgesic and neuroprotective effects during invasive procedures.

Three additional randomized controlled trials [10,24,25] assessed the effects of sucrose and breast milk both as standalone interventions and in combination with other non-pharmacological strategies. Talebi et al. [10] demonstrated that lullaby exposure, by mimicking the intrauterine environment, enhanced comfort and sleep quality; when combined with 24% sucrose, it resulted in greater reductions in heart rate and increased oxygen saturation during and after painful procedures compared to either intervention alone. Chang et al. [24] reported that non-nutritive sucking provided analgesic effects comparable to breastfeeding and sucrose, with enhanced physiological stability when combined with breastfeeding, although sucrose produced a more immediate reduction in crying. Cirik and Efe [25] found that the combination of breast milk and swaddling yielded the greatest analgesic effect, although all evaluated interventions independently improved heart rate and oxygen saturation.

Collectively, these findings indicate that both sucrose and breast milk, administered alone or in combination with complementary non-pharmacological strategies, effectively modulate pain responses in neonates, supporting their integration into multimodal pain management approaches in clinical practice.

## 4. Discussion

Overall, the findings of this systematic review confirm that both sucrose and breast milk are safe and effective non-pharmacological interventions for neonatal pain management, though they differ in their mechanisms of action and the benefits they provide.

Sucrose is characterized by its rapid and practical analgesic effect. Activation of sweet taste receptors triggers endogenous opioid release, providing immediate relief during painful procedures [34]. This makes sucrose particularly useful in situations where maternal presence is limited or direct access to the neonate is not possible [16]. In contrast, breast milk not only attenuates pain perception but also enhances overall neonatal well-being, supports physiological regulation, strengthens mother–infant bonding, and provides nutritional benefits, making it the preferred option whenever direct administration is feasible [22].

The efficacy of breast milk is further enhanced when combined with maternal sensory stimuli, such as olfactory and gustatory cues, skin-to-skin contact, or maternal heartbeat sounds [35]. Similarly, sucrose analgesia can be potentiated when paired with complementary interventions, including non-nutritive sucking or cooing [36]. Integrating these strategies optimizes both physiological parameters (e.g., heart rate and oxygen saturation) and behavioral indicators (e.g., crying duration and intensity), resulting in more comprehensive and sustained analgesia [37]. However, in contexts where simultaneous interventions are not feasible, individual administration of either sucrose or breast milk remains highly effective and safe, reinforcing their utility as first-line tools in neonatal pain management [24]. These findings highlight the importance of adapting intervention choice and combination to the clinical context, available resources, and individual characteristics of the neonate, thereby maximizing analgesic efficacy and promoting family-centered, humanized care.

The studies by Sen and Manav [29], Nimbalkar et al. [28], and Ghaemmaghami et al. [26] consistently show that both kangaroo care and 24% sucrose administration improve neonatal physiological responses during painful procedures, reducing heart rate and stabilizing oxygen saturation. However, these authors report superior analgesic effects with sucrose, likely attributable to its rapid activation of lingual sweet taste receptors and consequent endogenous opioid release. Nevertheless, kangaroo care provides complementary benefits, including thermoregulation, promotion of breastfeeding, and strengthening of the affective bond, thus solidifying its role within a comprehensive neonatal care strategy [38].

The formulation and mode of sucrose administration also influence analgesic efficacy. Bresesti et al. [6] demonstrated that sucrose gel promotes more intensive sucking, activating the parasympathetic system and producing immediate pain relief, suggesting that formulation can modulate analgesic response, especially when breastfeeding is not feasible. Similarly, Modaresi et al. [27] highlight that olfactory cues from breast milk can generate analgesia comparable to a 24% sucrose solution, emphasizing the importance of sensory signals in neonatal pain modulation.

When considered individually, both breast milk and sucrose effectively reduce neonatal pain. Experts report that breast milk confers additional benefits, including nutrition and mother–infant bonding, justifying its preferential use when direct administration is possible [39]. This observation is supported by subsequent studies [16,40], which demonstrated that both direct and donated breast milk reduce crying, heart rate, and pain scores, with direct breastfeeding providing superior analgesic effects compared to administration via syringe or pacifier.

Dosage and timing are critical for sucrose efficacy. Tanyeri-Bayraktar et al. [30] identified 0.2 mL/kg of 24% sucrose as the minimum effective dose, while Yaprak et al. [32] reported maximal analgesic effects at 2–3 min post-administration. These findings are consistent with prior evidence [41], supporting the use of small, rapidly acting doses for short-term procedural comfort. However, emerging evidence has raised concerns regarding the potential neurodevelopmental consequences of repeated sucrose exposure in very preterm infants. Recent clinical data in children born very preterm showed that cumulative neonatal pain exposure remained associated with increased internalizing behaviors at 18 months of corrected age, while sucrose administration did not mitigate these adverse behavioral outcomes [42]. In parallel, translational rodent studies have reported that repetitive early-life sucrose exposure may be associated with alterations in brain structure, including reductions in regional white matter and limbic volumes, as well as impairments in memory-related outcomes, despite short-term analgesic benefits [12]. Although these findings do not establish causality and should be interpreted considering important species and developmental differences, they highlight the need for caution regarding repeated sucrose administration during critical periods of brain development. Therefore, further research is warranted to clarify long-term safety, optimize dosing schedules, and identify strategies that effectively balance procedural analgesia with neurodevelopmental protection.

For breast milk, the studies by Taylar and Karakoc [31] and Cakirli and Acikgoz [23] show that direct breastfeeding combined with maternal sensory stimuli (e.g., heartbeat or milk odor) enhances analgesia and emotional bonding, whereas administration via syringe is less effective. Bulut et al. [22] further demonstrate that breast milk reduces cortical activity associated with pain, indicating that its analgesic effects extend beyond behavioral indicators such as crying.

Evidence from combined interventions highlights the additive benefits of multimodal approaches. Talebi et al. [10] found that cooing combined with 24% sucrose significantly decreased heart rate and improved oxygen saturation during painful procedures, exceeding the effects of either intervention alone. Chang et al. [24] reported that non-nutritive sucking provides analgesia comparable to breastfeeding and sucrose, with enhanced physiological stability when combined with breastfeeding, although sucrose produced more immediate reductions in crying. Cirik and Efe [25] demonstrated that combining breastfeeding with swaddling maximizes comfort and pain reduction. Recent studies [36,43] further support the safety and efficacy of complementary interventions, such as non-nutritive sucking and swaddling, whether used alone or in combination with breast milk or sucrose.

Accurate measurement of neonatal pain requires validated scales and trained assessors. This emphasizes that objective evaluation of physiological and behavioral parameters is essential to ensure reliability and comparability across studies. Heterogeneity in pain assessment tools and the inherent subjectivity of some indicators highlight the need for standardization in future research [44,45].

In summary, the evidence confirms that both sucrose and breast milk are effective analgesic strategies, each offering complementary advantages. Integration of multiple interventions, careful attention to sucrose dosage and formulation, and direct administration of breast milk can enhance analgesia and overall neonatal well-being. Nonetheless, further studies are required to establish standardized protocols, including optimal dosing, duration of effect, and combination of non-pharmacological methods.

### 4.1. Limitations

Despite the favorable findings, several limitations of this review and the included studies should be acknowledged. First, many of the trials involved small sample sizes, which may limit the statistical power and generalizability of the results.

Additionally, the restricted availability of family members in certain NICU settings limits the implementation of mother-centered interventions, such as direct breastfeeding or kangaroo care, potentially affecting the comparative assessment of analgesic methods. Variability in the execution of interventions by different practitioners introduces further heterogeneity, and differences in pain assessment by multiple observers increase the potential for measurement bias, even when validated scales are employed.

In addition, the methodological assessment using JBI and ROB-2 tools revealed important variability in study quality. While several trials demonstrated high methodological rigor, others showed limitations in randomization, allocation concealment, and blinding of participants, personnel, and outcome assessors. This heterogeneity in risk of bias across studies may have influenced the consistency and precision of the pooled evidence and should be considered when interpreting the overall conclusions of this review.

Finally, the absence of standardized protocols for non-pharmacological interventions complicates direct comparisons across studies and highlights the need for uniform, evidence-based institutional guidelines.

### 4.2. Implications for Clinical Practice

The findings of this review carry clear implications for clinical practice in the NICU. Both sucrose and breastfeeding are low-cost, safe, and readily implementable interventions that can be systematically incorporated into neonatal pain management protocols. Selection between methods should consider factors such as maternal availability, gestational age, and type of procedure, with direct breastfeeding prioritized whenever feasible due to its nutritional and affective benefits [46].

Complementary techniques, including cooing, non-nutritive sucking, and swaddling, can further enhance analgesic efficacy when combined with sucrose or breast milk, providing flexible strategies adaptable to diverse clinical contexts [15,43]. Implementation of these measures supports more humanized, family-centered care; reduces reliance on pharmacological agents; and minimizes potential drug-related adverse effects. Furthermore, the subject remains highly relevant to the field due to the limited number of safe and effective interventions available for neonatal pain management. Effective pain reduction strategies may help decrease the social burden on parents and could potentially influence neurodevelopmental outcomes and comorbidities associated with prematurity.

Finally, comprehensive training of healthcare personnel (especially nurses and midwives) and the adoption of evidence-based, protocolized guidelines are essential to maximize intervention effectiveness, ensure consistent application, and maintain high-quality care for neonates during painful procedures [19].

## 5. Conclusions

The evidence reviewed confirms that both sucrose and breast milk are highly effective non-pharmacological strategies for managing pain in neonates, demonstrating their clinical utility across a range of common painful procedures in NICUs. Breast milk provides significant analgesic effects while simultaneously promoting mother–infant bonding, skin-to-skin contact, and delivering nutritional and immunological benefits. Sucrose offers rapid, safe, and practical pain relief, particularly in situations where direct breastfeeding is not feasible.

When used individually, both interventions effectively reduce heart rate, stabilize oxygen saturation, and decrease crying duration, reflecting their impact on both physiological and behavioral responses to pain. Moreover, when combined with complementary non-pharmacological techniques, such as kangaroo care, non-nutritive sucking, or cooing, a synergistic effect is observed, producing greater analgesia than any single method alone.

These findings strongly support the integration of sucrose and breast milk into standardized clinical protocols, promoting humanized, accessible, and cost-effective practices that optimize neonatal well-being and safety during invasive procedures. Finally, the review emphasizes the need for further research to refine dosage regimens, evaluate optimal combinations of techniques, and establish evidence-based application criteria tailored to gestational age and individual clinical conditions.

## Figures and Tables

**Table 1:** Search strategy results from the databases.

Database	Search Strategy	Filter	Results
PubMed	(sucrose OR breastfeeding OR breastfed OR “breast milk”) AND (pain* OR “physical suffering” OR ache*) AND (neonate* OR newborn* OR “newborn infant”)	Date: 2019–2026	857
Scopus	(sucrose OR breastfeeding OR breastfed OR “breast milk”) AND (pain* OR “physical suffering” OR ache*) AND (neonate* OR newborn* OR “newborn infant”)	Date: 2019–2026	1111
CINAHL	(sucrose OR breastfeeding OR breastfed OR “breast milk”) AND (pain* OR “physical suffering” OR ache*) AND (neonate* OR newborn* OR “newborn infant”)	Date: 2019–2026	498
Web of Science	(sucrose OR breastfeeding OR breastfed OR “breast milk”) AND (pain* OR “physical suffering” OR ache*) AND (neonate* OR newborn* OR “newborn infant”)	Date: 2019–2026	1246

**Table 2:** JBI Critical Appraisal Checklist for Randomized Controlled Trials.

Article	Q1	Q2	Q3	Q4	Q5	Q6	Q7	Q8	Q9	Q10	Q11	Q12	Q13	TOTAL
Bresesti et al., 2021 [6]	No	Unclear	Yes	No	No	No	No	Yes	Yes	Yes	Yes	Yes	No	6/13
Bulut et al., 2024 [22]	Yes	Yes	Yes	No	No	Yes	No	Yes	Yes	Yes	Yes	Yes	Yes	10/13
Cakirli & Acikgoz, 2021 [23]	Yes	Unclear	Yes	Unclear	No	No	No	Yes	Yes	Yes	Yes	Yes	Yes	9/13
Chang et al., 2020 [24]	No	Unclear	Yes	No	No	No	No	Yes	Yes	Yes	Yes	Yes	No	6/13
Cirik & Efe, 2020 [25]	Yes	Yes	Yes	No	No	Yes	Yes	Yes	Yes	Yes	Yes	Yes	Yes	12/13
Ghaemmaghami et al., 2024 [26]	Yes	Unclear	Yes	No	No	Yes	Yes	Yes	Yes	Yes	Yes	Yes	Yes	11/13
Modaresi et al., 2024 [27]	No	No	Yes	No	No	Unclear	Yes	Yes	Yes	Yes	Yes	Yes	No	7/13
Nimbalkar et al., 2020 [28]	Yes	Yes	Yes	No	No	Yes	Yes	Yes	Yes	Yes	Yes	Yes	Yes	12/13
Sen & Manav, 2020 [29]	Yes	Yes	Yes	No	No	Unclear	Yes	Yes	Yes	Yes	Yes	Yes	Yes	11/13
Talebi et al., 2022 [10]	Yes	Yes	Yes	No	No	Yes	Yes	Yes	Yes	Yes	Yes	Yes	Yes	12/13
Tanyeri-Bayraktar et al., 2019 [30]	Yes	Unclear	No	No	Yes	Yes	Yes	Yes	Yes	Yes	Yes	Yes	Yes	10/13
Tavlar & Karakoc, 2022 [31]	Yes	Unclear	Yes	No	No	No	Yes	Yes	Yes	Yes	Yes	Yes	Yes	9/13
Yaprak et al., 2023 [32]	Yes	Yes	Yes	No	Yes	Yes	Yes	Yes	Yes	Yes	Yes	Yes	Yes	12/13

Legend/Key: Q1–Q13: Questions from the RCT assessment checklist. True randomization used? Allocation concealed? Baseline group similarity? Participants blinded? Personnel delivering treatment blinded? Outcome assessors blinded? Groups treated identically except for intervention? Complete follow-up and handling of differences? Analysis by assigned groups (intention-to-treat)? Outcomes measured the same way across groups? Outcomes measured reliably? Appropriate statistical analysis used? Trial design appropriate, deviations accounted for? Response options for questions: Yes—criterion clearly met; No—criterion clearly not met; Unclear—insufficient information to judge.

**Table 3:** Risk of Bias assessment using ROB-2 tool.

Study (Author, Year)	D1. Bias from Randomization	D2. Bias from Deviations	D3. Bias from Missing Data	D4. Bias in Measurement	D5. Bias in Reporting	Overall Risk of Bias
Bresesti et al., 2021 [6]	High risk	Some concerns	Low risk	Some concerns	Low risk	High risk
Bulut et al., 2024 [22]	Low risk	Some concerns	Low risk	Low risk	Low risk	Some concerns
Cakirli & Acikgoz, 2021 [23]	Low risk	Low risk	Low risk	Some concerns	Low risk	Some concerns
Chang et al., 2020 [24]	Some concerns	Some concerns	Low risk	High risk	Some concerns	High risk
Cirik & Efe, 2020 [25]	Low risk	Low risk	Low risk	Low risk	Low risk	Low risk
Ghaemmaghami et al., 2024 [26]	Low risk	Low risk	Low risk	Low risk	Low risk	Low risk
Modaresi et al., 2024 [27]	High risk	Some concerns	Low risk	Some concerns	Low risk	High risk
Nimbalkar et al., 2020 [28]	Low risk	Low risk	Low risk	Low risk	Low risk	Low risk
Sen & Manav, 2020 [29]	Low risk	Some concerns	Low risk	Some concerns	Low risk	Some concerns
Talebi et al., 2022 [10]	Low risk	Low risk	Low risk	Low risk	Low risk	Low risk
Tanyeri-Bayraktar et al., 2019 [30]	Low risk	Low risk	Low risk	Low risk	Low risk	Low risk
Tavlar & Karakoc, 2022 [31]	Low risk	Low risk	Low risk	High risk	Low risk	High risk
Yaprak et al., 2023 [32]	Low risk	Low risk	Low risk	Low risk	Low risk	Low risk

**Table 4:** Characteristics of the included studies.

Author, Year, Country	Methodology, Measuring Instrument	Objective
(Sen & Manav, 2020) Istanbul, Turkey [29]	Prospective randomized controlled trial included 64 preterm infants hospitalized in the intensive care unit. 32 patients received kangaroo care. 32 patients received oral sucrose. Heart rate, oxygen saturation, and pain score were measured. Measuring instrument: Premature Infant Pain Profile (PIPP)	To compare the effects of kangaroo and oral sucrose in pain relief in preterm infants during heel puncture.
(Tavlar & Karakoc, 2022) Istanbul, Turkey [31]	Randomized, experimental, controlled trial with a sample of 90 newborns. The distribution of the sample was as follows: 30 patients assigned to breastfeed; 30 patients assigned to the smell of breast milk; 30 patients assigned to the mother’s heartbeat group. Measuring instrument: Astrid Lindgren Children’s Hospital Pain Scale (ALPS-Neo)	To assess the effect of breastfeeding, the smell of breast milk, and the sound of the mother’s heartbeat on the level of pain in newborns.
(Bresesti et al., 2021) Milan, Italy [6]	This was a prospective, randomized, controlled trial. The study was conducted as a comparative effectiveness investigation including 195 neonates who were randomly assigned into 3 groups: Breastfeeding (*n =* 65); Administered 2 mL of 24% liquid sucrose with non-nutritive suction (silicone pacifier) through a syringe (*n =* 65); Administered 24% sucrose gel with a 2 mL syringe with non-nutritive suction (silicone pacifier) (*n =* 65). Measurement instrument: Neonatal Infants Pain Scale (NIPS)	To compare the efficacy of non-pharmacological measures for pain control in neonates: 24% liquid sucrose vs. 24% gel sucrose vs. breastfeeding.
(Nimbalkar et al., 2020) Anand, Gujarat, India [28]	A total of 100 infants were included in this trial between 28 and 36 weeks of gestation. The chosen participants were randomly assigned into two groups: Skin-to-skin contact was administered 15 min before heel puncture and sucrose 2 min before the second puncture (*n =* 50); Administered 24% sucrose 2 min before the first puncture and 15 min prior to the second puncture skin-to-skin contact (*n =* 50). Measuring instrument: Premature Infant Pain Profile (PIPP)	To compare skin-to-skin care and oral sucrose for the control of premature neonatal pain.
(Talebi et al., 2022) Babol, Iran [10]	A total of 60 neonates from the hospital were included in this prospective control study, who randomly divided into 4 groups of 15 patients each. Patients were swaddled prior to the procedure. Patients were given sucrose. Patients were swaddled and sucrose was administered simultaneously. In the control group, no measures were applied to perform the procedure. Measuring instrument: Premature Infant Pain Profile (PIPP)	To investigate the effect of concurrent use of swaddling and sucrose on pain intensity before a procedure in neonatal patients.
(Ghaemmaghami et al., 2024) Shiraz, Iran [26]	This was a randomized prospective control trial study. The sample consisted of 66 neonates who were assigned into 3 groups by random assignment, with a total of 22 newborns in each. The patients received kangaroo methods during the procedure. Patients were given 24% sucrose during the procedure. The control group received routine care without any intervention. Measuring instrument: Neonatal Infants Pain Scale (NIPS)	To compare the effects of oral sucrose and kangaroo care on selected physiological variables and pain scores resulting from venipuncture.
(Tanyeri-Bayraktar et al., 2019) Istanbul, Turkey [30]	This was a prospective, randomized, double-blind study. A total of 129 infants were included and randomly divided into 2 groups: 65 patients received 0.2 mL/kg of 24% sucrose before venipuncture; 64 patients received 0.5 mL/kg of 24% sucrose before venipuncture. Measuring instrument: Bernese Pain Scales for Neonates (BPSN)	To compare the efficacy of two different doses of sucrose during venipuncture in neonates.
(Yaprak et al., 2023) Ankara, Turkey [32]	A prospective, randomized, controlled study was conducted. There were 54 participating newborns, who were randomly assigned into 2 groups. 24% sucrose was administered orally 2 min before heel puncture. 24% sucrose was administered with no interval before heel puncture. Measuring instrument: Premature Infant Pain Profile-Revised (PIPP-R)	To assess the availability of sucrose analgesia in states of emergency due to minor procedural pain by eliminating the 2 min interval before heel puncture in preterm infants.
(Chang et al., 2020) Los Angeles, USA [24]	A total of 226 neonatal patients were randomly assigned to one of the 5 intervention groups, the last of which was a control group: 45 patients in the breastfeeding group; 42 patients in the 24% oral sucrose group; 51 patients in the non-nutritive sucking (pacifier) group; 38 patients in the skin-to-skin contact group; 50 patients in the control group. The newborns were placed in a crib in a supine position and covered with blankets. Measurement instrument: Neonatal Pain, Agitation and Sedation Scale (NPASS)	To compare the analgesic effect of 4 non-pharmacological interventions (breastfeeding, 24% sucrose, non-nutritive suction, and skin-to-skin contact) in term infants who underwent heel pricks.
(Cakirli & Acikgoz, 2021) Eskisehir, Turkey [23]	This experimental, randomized, controlled study divided a total of 90 patients into 3 groups: 30 patients exposed to the smell of breast milk from their own mother; 30 patients exposed to the smell of breast milk from another mother; 30 patients in the control group. Measuring instrument: Neonatal Pain, Agitation and Sedation Scale (NPASS)	To compare the effect of the smell of breast milk from the baby’s own mother versus that of another mother’s milk for pain reduction in newborns.
(Cirik & Efe, 2020) Istanbul, Turkey [25]	Prospective, controlled and randomized study that included 187 infants who were divided into 6 groups: Routine care (*n =* 33); Lullaby method (*n =* 30); Facilitated involvement (*n =* 32); Expressed breast milk (*n =* 31); Lullaby and expressed breast milk (*n =* 30); Facilitated wrapping and expressed breast milk (*n =* 31). Measuring instrument: Premature Infant Pain Profile (PIPP)	To compare the effects of breast milk, swaddling the baby, facilitated swaddling, expressed breast milk and swaddling, breast milk and facilitated swaddling, and routine care.
(Bulut et al., 2024) Istanbul, Turkey [22]	Prospective, controlled and randomized study that included 90 infants who were divided into 2 groups: 45 patients received 2 mL of oral breast milk; 45 patients in the control group did not receive any intervention. Measuring instrument: Neonatal Pain, Agitation and Sedation Scale (NPASS)	To investigate the effects of breast milk on cortical pain response and behavioral response in neonates during the heel puncture procedure.
(Modaresi et al., 2024) Sari, Iran [27]	Prospective randomized controlled trial that included 99 term infants who were randomly divided into 3 groups: Administration of 24% sucrose (*n =* 34); Exposed to smell of breast milk (*n =* 31); Exposed to breast milk flavor (*n =* 34). Measurement instrument: Neonatal Infant Acute Pain Assessment Scale (NIAPAS)	To compare the effect of 24% sucrose with smelling and tasting breast milk on pain intensity during venipuncture in neonates.

**Table 5:** Outcomes reported in the included studies.

Author, Year, Country	Results
(Sen & Manav, 2020) Istanbul, Turkey [29]	Both kangaroo care and oral sucrose administration were found to significantly decrease the level of pain in neonates during procedures. The PIPP values were lower in the kangaroo group compared to the sucrose group, resulting in the conclusion that in addition to reducing pain more than when sucrose is used, the combination favors well-being, offering additional benefits such as stabilization of heart rate and oxygen saturation, and the strengthening of the bond between mother and child.
(Tavlar & Karakoc, 2022) Istanbul, Turkey [31]	Newborns whose intervention was the smell of breast milk had a higher level of pain and stress compared to those patients who belonged to the heartbeat sound group who had milder pain. Although the mother’s heartbeat method is effective, breastfeeding is the most effective method in painful processes in the neonatal patient; it reduces pain and stress almost completely.
(Bresesti et al., 2021) Milan, Italy [6]	All the interventions analyzed were shown to be effective in reducing neonatal pain. The administration of 24% liquid sucrose was shown to be less effective than breastfeeding. The 24% sucrose gel with non-nutritive suction had a lower probability of pain compared to liquid sucrose. In short, although all interventions are effective, the combination of 24% sucrose gel with non-nutritive suction could be a valid alternative when breastfeeding is not possible.
(Nimbalkar et al., 2020) Anand, Gujarat, India [28]	Both interventions proved to be effective in reducing pain in neonates, as shown by the PIPP scores, which showed significant decreases in both groups. The PIPP score at minute 0 (just before heel puncture) and at 5 min after the intervention was lower in the sucrose group compared to the skin-to-skin group. Sucrose was well tolerated by all patients in the group with no immediate side effects observed. The heart rate component in the PIPP score was the only one that was lower in the skin-to-skin group, but it was not statistically significant.
(Talebi et al., 2022) Babol, Iran [10]	Non-pharmacological pain management with wrap and sucrose has been shown to be the most effective method and is the most convenient, practical and cheapest for painful procedures in neonates. Therefore, for better pain management, it is recommended to use the combined sucrose and wrapping method instead of routine methods like using sucrose alone.
(Ghaemmaghami et al., 2024) Shiraz, Iran [26]	Follow-up tests revealed that, during blood draw, mean respiration rate, heart rate, oxygen saturation, and pain score in the oral sucrose group decreased significantly compared to the kangaroo group, with sucrose administration being more effective than kangaroo methods. It is important to highlight the similarity of the effects of sucrose and breastfeeding.
(Tanyeri-Bayraktar et al., 2019) Istanbul, Turkey [30]	Six patients were excluded from the study for different reasons. After this, no statistically significant difference was found in BPSN scores between the groups (*p* > 0.05). There was also no statistically significant difference with respect to blood glucose levels (*p* > 0.05). The results therefore showed that 0.2 mL/kg of 24% sucrose could be the minimally sufficient dose to relieve pain during painful procedures in neonates.
(Yaprak et al., 2023) Ankara, Turkey [32]	No significant differences in PIPP-R scores were observed between the groups that received 24% sucrose orally with and without a time interval before the procedure. It is therefore concluded that, after the administration of sucrose, a waiting period of 2 min is not necessary even in emergency situations as it is a safe and effective method for these patients. It should be noted, however, that sucrose has its maximum analgesic efficacy in neonates between 2 and 3 min after its administration.
(Chang et al., 2020) Los Angeles, USA [24]	The 5 interventions were performed and analyzed using the NPASS. The results showed that the analgesic effect of oral sucrose is statistically superior to skin-to-skin contact. No differences were observed between the other intervention groups. The 24% oral sucrose treatment was the most effective intervention in reducing crying time compared to the other non-pharmacological measures. It was therefore concluded that oral sucrose has an immediate analgesic effect, which is effective in reducing agitation and crying in neonatal patients.
(Cakirli & Acikgoz, 2021) Eskisehir, Turkey [23]	The most effective method for reducing pain was shown to be the smell of the baby’s own mother’s breast milk, which showed a lower score on the N-PASS compared to the other groups. Regarding the total crying time, similar results were found between the two groups that used breast milk, which differed from the results of the control group, which had a significantly higher value.
(Cirik & Efe, 2020) Istanbul, Turkey [25]	According to the results obtained, the lullaby method, the expressed breast milk method and the facilitated restraint method have good analgesic effects, reducing pain during invasive procedures such as the insertion of an orogastric tube. The combination of the lullaby and expressed breast milk methods was the most effective method as it produced a significantly lower score in the pain profile compared to the other groups.
(Bulut et al., 2024) Istanbul, Turkey [22]	An infrared device was used to monitor regional cerebral oxygen saturation, and the N-PASS was used to assess pain. It was concluded that the score from the scale and the duration of infant crying were lower in the group that received breast milk compared to the control group. There was no significant difference between the two groups with respect to oxygen saturation and heart rate during and after the procedure; none of the measures improved these parameters.
(Modaresi et al., 2024) Sari, Iran [27]	The use of the smell and taste of milk is a highly efficient method, which was almost comparable to the 24% sucrose solution. It is important to mention that the smell of breast milk was more effective compared to its taste, as the babies were able to detect the smell of their own mothers and respond to it, reducing the effects of painful procedures on the newborn.

## Data Availability

All data available within this article.

## Abbreviations

The following abbreviations are used in this manuscript:

WHO	World Health Organization
NICU	Neonatal Intensive Care Unit
CRIES	Crying, Requires O_2_, Increased vitals, Expression, Sleeplessness
PIPP	Premature Infant Pain Profile
PIPP-R	Premature Infant Pain Profile–Revised
NIPS	Neonatal Infant Pain Scale
ALS-Neo	Assessment of Pain in Neonates Scale
BPSN	Bernese Pain Scale for Neonates
NIAPAS	Neonatal Infant Acute Pain Assessment Scale
N-PASS	Neonatal Pain, Agitation and Sedation Scale
RCT	Randomized controlled trial
PRISMA	Preferred Reporting Items for Systematic Reviews and Meta-Analyses
PICO	Population, Intervention, Comparator, Outcome
WOS	Web of Science
JBI	Joanna Briggs Institute
